# Total Plasma Protein in Very Preterm Babies: Prognostic Value and Comparison with Illness Severity Scores

**DOI:** 10.1371/journal.pone.0062210

**Published:** 2013-04-16

**Authors:** Silvia Iacobelli, Francesco Bonsante, Catherine Quantin, Pierre-Yves Robillard, Christine Binquet, Jean-Bernard Gouyon

**Affiliations:** 1 Néonatologie, Réanimation Néonatale et Pédiatrique, CHU La Réunion-Site Sud, Saint Pierre, France; 2 Centre d'Etudes Périnatales de l'Océan Indien, CHU La Réunion–Site Sud, Saint Pierre, France; 3 CHRU, Service de Biostatistique et d'Informatique Médicale, Dijon, France; 4 INSERM U866, Dijon, Université de Bourgogne, Dijon, France; 5 INSERM CIE1, Dijon, France; CHRU Dijon, Centre d'Investigation Clinique-Epidémiologie Clinique/Essais Cliniques, Dijon, France; Université de Bourgogne, Dijon, France; Hôpital Robert Debré, France

## Abstract

**Objective:**

We aimed to investigate the predictive value for severe adverse outcome of plasma protein measurements on day one of life in very preterm infants and to compare total plasma protein levels with the validated illness severity scores CRIB, CRIB-II, SNAP-II and SNAPPE-II, regarding their predictive ability for severe adverse outcome.

**Methods:**

We analyzed a cohort of infants born at 24–31 weeks gestation, admitted to the tertiary intensive care unit of a university hospital over 10.5 years. The outcome measure was “severe adverse outcome” defined as death before discharge or severe neurological injury on cranial ultrasound. The adjusted odd ratio (aOR) and 95% confidence interval (95% CI) of severe adverse outcome for hypoproteinemia (total plasma protein level <40 g/L) was calculated by univariate and multivariate analyses. Calibration (Hosmer-Lemeshow goodness-of-fit) was performed and the predictive ability for severe adverse outcome was assessed for total plasma protein and compared with CRIB, CRIB-II, SNAP-II and SNAPPE-II, by calculating receiver operating characteristic (ROC) curves and their associated area under the curve (AUC).

**Results:**

761 infants were studied: 14.4% died and 4.1% survived with severe cerebral ultrasound findings. The aOR of severe adverse outcome for hypoproteinemia was 6.1 (95% CI 3.8–9.9). The rank order for variables, as assessed by AUCs and 95% CIs, in predicting outcome was: total plasma protein [0.849 (0.821–0.873)], SNAPPE-II [0.822 (0.792–0.848)], CRIB [0.821 (0.792–0.848)], SNAP-II [0.810 (0.780–0.837)] and CRIB-II [0.803 (0.772–0.830)]. Total plasma protein predicted severe adverse outcome significantly better than CRIB-II and SNAP-II (both p<0.05). Calibration for total plasma protein was very good.

**Conclusions:**

Early hypoproteinemia has prognostic value for severe adverse outcome in very preterm, sick infants. Total plasma protein has a predictive performance comparable with CRIB and SNAPPE-II and greater than other validated severity scores.

## Introduction

Advances in perinatal care have resulted in an improvement in the survival rate of very low birth weight infants (VLBWI) as well as in a decrease of the disability rate in VLBW survivors [1], [2]. Amidst all the progress made over decades, neonatal outcome has been benefiting from the development and the use of illness severity scores, which have permitted quality of care evaluation, risk adjustment comparisons in benchmarking studies, management and resource implementation. The Clinical Risk Index for Babies (CRIB) [3], its revised version (CRIB-II) [4], the Score for Neonatal Acute Physiology II (SNAP-II) and the SNAP Perinatal Extension II (SNAPPE-II) [5] are the most widely used scores to estimate illness severity and in-hospital mortality risk in the neonatal intensive care unit (NICU).

Some authors have underlined that risk adjustment using these scores is imperfect because additional perinatal factors may significantly influence VLBWI survival [6]. Others have noted the deterioration over time in their predictive performance, due to incremental improvement of care [7]. Actually, the relationship between physiological status and mortality risk may change as preventive interventions, monitoring strategies and new treatment protocols are introduced. Infants with similar illness severity scores may differ for their risk of death [6] and a better understanding of all the perinatal factors influencing mortality remains a meaningful challenge for neonatologists.

Another point at issue is that, even if survival has progressively improved especially after extremely preterm birth, the high rate of disability in survivors is still a concern [8]. So, for the clinician involved in assessing the initial risk of high vulnerable neonatal populations, death is not the only important adverse outcome, and a primary goal is also to quantify the risk of severe brain damage.

Recently we reported, for the first time, that hypoproteinemia (total protein level of less than 40 g/L) on day 1 of life is an independent factor associated with severe adverse outcome (SAO), defined as in-hospital death or severe neurological injury on cranial ultrasound, in a large sample of critically ill preterm babies [9]. In order to further investigate the clinical interest of this finding, we performed a study with the following objectives: 1) to confirm the prognostic value for SAO of hypoproteinemia in another population of VLBWI; 2) to compare total plasma protein levels alone with composite scores CRIB, CRIB-II, SNAP-II and SNAPPE-II, regarding their predictive ability for SAO in this population.

## Methods

### Ethics statement

This study was approved by the institutional medical research ethics committee (Comité de Protection des Personnes Sud-Ouest et Outre Mer III, authorization number 2012/36).

According to French legislation, written parental consent was not needed for this study.

### Design and study population

The study design was an observational cohort analysis of all the infants born between 24 and 31 weeks of gestational age (GA) and admitted within 12 hours of life to the tertiary NICU of Saint Pierre University Hospital (Reunion Island, France) during a 10.5 year period (1 January 2001 to 30 June 2011). Patients were excluded if they died within the first 12 hours after birth, if clinical data were incomplete, if plasma protein value on the first day of life was not available or if any of the items for calculating the CRIB, CRIB-II, SNAP-II and SNAPPE-II were missing.

### Data collection

Clinical data were drawn from the unit perinatal database, which has prospectively recorded demographic, gestational and perinatal variables of all mother-infant pairs since 2001. This recording of mother-infant clinical information was approved by the National Committee for data protection (Commission Nationale de l'Informatique et des Libertés, registration number 1620660). Information was collected at the time of delivery and at the time of infant hospital discharge and regularly audited by appropriately trained staff. For the purpose of this study, records have been validated and have been used anonymously. Available information in this data set included maternal age, parity and gravidity, pre-existing clinical and gynecological diseases, whether singleton or multiple pregnancy, obstetric history and illness, antenatal steroid administration, labor and delivery complications, mode of delivery (vaginal/caesarean section), outborn (transfer after birth, admitted into NICU within 12 hours of life), gender, GA, birth weight (BW), cord blood lactate concentration, 1 and 5 minute Apgar score, neonatal morbidities and neonatal death.

### Total protein values, CRIB, CRIB-II, SNAP-II and SNAPPE-II

Data on total protein values, CRIB, CRIB-II, SNAPP-II and SNAPPE-II were abstracted from the medical charts.

According to the unit guidelines for assessing fluid and electrolyte status in VLBWI, babies born less than 32 weeks of GA and hospitalized in our NICU had a blood sample at around 12 hours of life. The plasma total protein measurement provided by this blood sample was used for the purpose of this study.

Hypoproteinemia was defined as a total protein level of less than 40 g/L [9], [10].

CRIB was calculated as already described [3], using the six variables: BW, GA, presence of congenital malformations, maximum base excess in the first 12 hours of life, and minimum and maximum appropriate fraction of inspired oxygen –FiO_2_–(FiO_2_ min) and (FiO_2_ max) in the first 12 hours. CRIB-II was calculated using the variables BW, GA, gender, temperature and base excess on admission [4]. Following the original paper from Richardson et al [5], the six item score (urine output, lowest mean blood pressure, worst PaO_2_/FiO_2_ ratio, lowest pH, occurrence of seizures, lowest temperature) was used for SNAP-II. To this, BW, small for GA, Apgar at 5 minutes were added for calculating the SNAPPE-II [5]. The data collection window for these two scores was the first 12 hours after birth.

### Outcome measure

The outcome measure was SAO. This was defined as death or severe (grade 3 or 4) intraventricular hemorrhage or cystic periventricular leukomalacia occurring before infants' discharge from hospital. Intraventricular hemorrhage was graded at cerebral ultrasound according to Papile et al [11]. Cystic periventricular leukomalacia was defined by cranial ultrasound as an area of increased echogenicity of the periventricular white matter in acute phase which subsequently evolved into cystic lesion [12]. Cranial ultrasounds were routinely performed during the infant hospital stay by experienced examiners according to the following protocol: day 1, 7, 10, 15 and then at least every 2 weeks or more often as clinically indicated, until discharge.

### Statistical analysis

We assessed the prognostic value for SAO of total plasma protein in two sets of analyses.

In the first analysis, we estimated and compared the incidence of SAO in hypoproteinemic patients and in patients with normal protein values. The crude odd ratio (OR) and 95% confidence interval (95% CI) of SAO for hypoproteinemia were calculated. We then used backward stepwise logistic regression to obtain the adjusted OR for potential confounding variables. Univariate analysis was first performed and variables significant at a p value <0.10 were entered in the multivariate model. For this analysis continuous variables were categorized by cut-off values chosen as having the highest Youden index [13].

In the second analysis, discrimination-that is the ability to correctly predict SAO-was assessed for total plasma protein levels and compared with CRIB, CRIB-II, SNAP-II, SNAPPE-II, BW and GA discrimination, by calculating receiver operating characteristic (ROC) curves and their associated area under the curve (AUC), with 95% CI based on the observed values entered on a continuous scale [14]. An AUC value of 0.5 indicates no better than chance ability to discriminate and larger values indicate increasing ability. A value above 0.8 is considered good [15]. Calibration of total plasma protein levels, CRIB, CRIB-II, SNAP-II, SNAPPE-II, BW and GA was investigated using the Hosmer and Lemeshows (HL) goodness-of-fit test, which categorizes the observations into groups according to their predicted risk [15]. The numbers of predicted and observed outcomes within each of these groups are compared. A non-significant p value of the HL indicates an acceptable calibration.

Comparisons between groups were performed using χ^2^-test or Fisher's exact test for categorical variables; the ANOVA test was used for parametric variables and the Mann-Whitney *U* test for non-parametric continuous variables. Comparison of the AUCs was evaluated by the DeLong method [16]. All statistical analyses were carried out using the MedCalc. ver. 12.3.0.0 statistical software package (MedCalc Software Mariakerke, Belgium) and p values <0.05 were considered statistically significant.

## Results

From 1 January 2001 to 30 June 2011, 841 neonates born below 32 weeks of gestation were hospitalized in our NICU within the first 12 hours of life. Three babies died within 12 hours after birth, clinical data were missing or incomplete for 26, and total protein value was not obtainable for 51 infants. The items to calculate CRIB, CRIB-II, SNAP-II and SNAPPE-II were always available, so all the remaining 761 newborns were eligible for the analysis. Infants with missing data were similar in their characteristics and outcomes when compared to those included in the study (data not shown).

SAO was present in 140 patients (18.4% of the study population): 109 patients (14.4%) died and 31 (4.1%) survived with severe cerebral ultrasound findings. Table 1 shows antenatal characteristics of the study population, characteristics at birth and postnatal diseases.

**Table 1:** Population characteristics (n = 761 preterm infants<32 weeks gestation).

Antenatal variables	
Maternal age, mean±SD, y	27.7±6.8
Hypertensive Disease of Pregnancy, %	22.3
Maternal Diabetes, %	9.7
Maternal Body Mass Index>30 before pregnancy, %	16.0
Antenatal Steroids, %	69.6
Singleton Pregnancy, %	78.8

Population characteristics	
Birth Weight, mean±SD, g	1203±346
Gestational Age, mean±SD, wk	28.8±1.9
Small for Gestational Age, %	9.9
Male Gender, %	50.3
Apgar Score≤3 @ 1 minute, %	10.1
Caesarean Section, %	56.4
Outborn, %	2.0
Congenital malformations, %	2.7
Cord blood lactate concentration, mean±SD, mmol/L	4.0±3.5
CRIB, mean±SD	2.6±3.1
CRIB-II, mean±SD	6.2±3.4
SNAP-II, mean±SD	14.7±14.8
SNAPPE-II, mean±SD	20.7±20.2
Hypoproteinemia, %	24.0
RDS requiring invasive mechanical ventilation, %	59.4
Anemia at birth, %	15.6
Treated hypotension on day 1 of life, %	22.6
Treated Patent Ductus Arteriosus %	15.1
Necrotizing Enterocolitis, %	4.5
Bronchopulmonary Dysplasia @ 36 weeks, %	4.7

(CRIB) Clinical Risk Index for Babies; (CRIB-II) revised Clinical Risk Index for Babies;

(SNAP-II) Score for Neonatal Acute Physiology II; (SNAPPE-II) Score for Neonatal Acute Physiology Perinatal Extension II; (RDS) Respiratory Distress Syndrome.

Hypoproteinemia occurred in 24% of all patients. The blood sample was carried out at 14.7±5.3 (mean±SD) hours after birth. The rate of SAO was 52.5% in patients with hypoproteinemia and 7.6% in those with normal protein values (p<0.001; crude OR 13.3; 95% CI 8.7–20.4). The univariate procedure yielded 16 variables for inclusion into the multivariate logistic model: maternal age≥25 years, multiple pregnancy, time-frame, steroid administration, cesarean section delivery, outborn, male gender, gestational age, FiO_2_ max>40%, FiO_2_ min>23%, Apgar score≤3 at 1 minute, hypoproteinemia, cord blood lactate>4 mmol/L, congenital malformations, admission temperature≤35.1°C and anemia at birth (data not shown).

Results of the multivariate analysis are reported in table 2.

**Table 2:** Variables associated with severe adverse outcome at multivariate analysis in the study population (n = 761 preterm infants<32 weeks gestation).

Variables	OR	95% CI	P
Hypoproteinemia	6.1	3.8–9.9	<0.001
GA 24–25 versus 28–31 weeks	3.7	1.7–7.9	<0.001
GA 26–27 versus 28–31 weeks	3.1	1.8–5.1	<0.001
FiO_2_ max>40%	1.9	1.1–3.1	0.01
FiO_2_ min>23%	3.1	1.6–5.8	0.001
Apgar Score≤3 @1 minute of life	2.5	1.4–4.8	0.003

(GA) Gestational age; FiO_2_ (Fraction of inspired oxygen)

There was a significant linear association between GA and plasma protein levels, (r^2^ 0.23, p<0.001, data not shown). However at the multivariate analysis both were independent factors strongly associated with SAO. The adjusted OR of SAO for hypoproteinemia after correcting for the confounding variables was 6.1 (95% CI 3.8–9.9).

**Figure 1:**
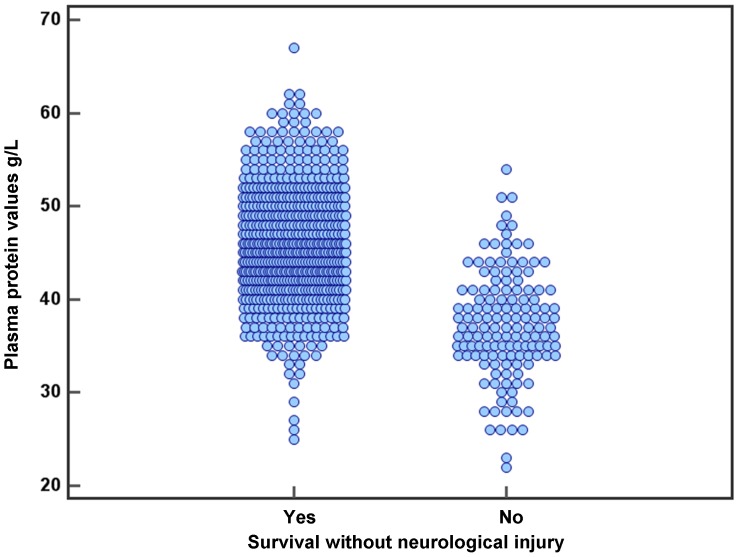
Scattergram demonstrating the total plasma protein values in infants with severe adverse outcome compared to those who survived without severe neurological injury.

Figure 1 shows the scattergram illustrating the total plasma protein values in infants with SAO compared to those who survived without severe neurological injury.

**Figure 2:**
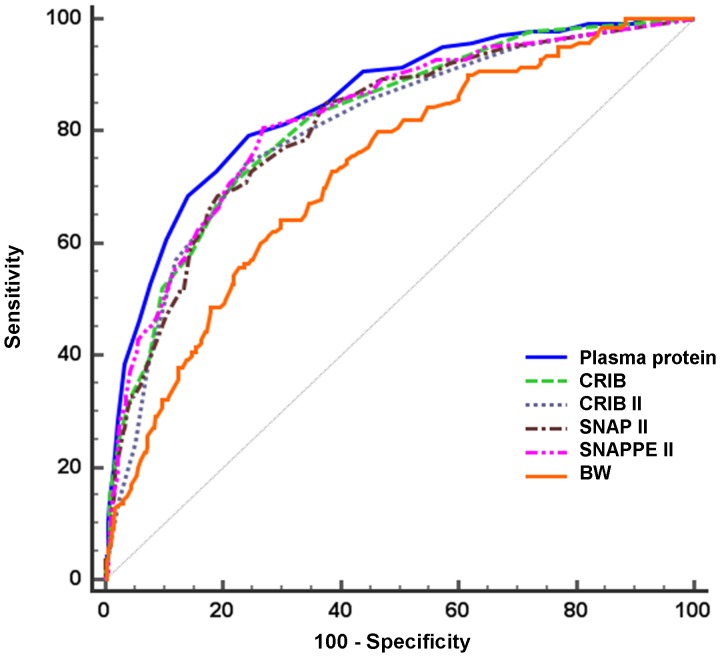
Receiver operating characteristic curves of plasma protein levels, CRIB, CRIB-II, SNAP-II, SNAPPE-II and birth weight (BW).

ROC curves for total plasma protein levels, illness severity scores and BW are reported in Figure 2.

The rank order for variables, as assessed by AUCs and 95% CIs in predicting SAO, was plasma protein levels, SNAPPE-II, CRIB, SNAP-II and CRIB-II. Plasma protein levels predicted SAO significantly better than CRIB-II and SNAP-II, while no significant difference was seen with CRIB and SNAPPE-II. Calibration for plasma protein levels was very good (p = 0.79 at the HL goodness-of-fit test, result shown in table 3).

**Table 3:** Predictive accuracy of plasma protein levels, CRIB, CRIB-II, SNAP-II, SNAPPE-II, birth weight (BW) and gestational age (GA) in identifying severe adverse outcome in the study population.

Variables	AUC±95% CI	ROC curve comparison*	p**
(1) Plasma protein levels	0.849 (0.821–0.873)	p<0.05 vs (3), (5), (6), (7)	0.73
(2) CRIB	0.821 (0.792–0.848)	p<0.05 vs (7)	0.01
(3) CRIB-II	0.803 (0.772–0.830)	p<0.05 vs (7)	0.20
(4) SNAPPE-II	0.822 (0.792–0.848)	p<0.05 vs (7)	0.26
(5) SNAP-II	0.810 (0.780–0.837)	p<0.05 vs (7)	0.47
(6) GA	0.784 (0.753–0.813)	p<0.05 vs (7)	0.65
(7) BW	0.727 (0.694–0.759)		0.56

Result of receiver operating characteristic curves analyses and comparison in performances.

* p value for DeLong test comparing AUCs

** p value for Hosmer-Lemeshow goodness-of-fit test.

(CRIB) Clinical Risk Index for Babies; (CRIB-II) revised Clinical Risk Index for Babies;

(SNAP-II) Score for Neonatal Acute Physiology II; (SNAPPE-II) Score for Neonatal Acute Physiology Perinatal Extension II; (GA) Gestational Age; (BW) Birth Weight.

Table 3 shows the results of AUCs curves, AUCs comparison and calibration for all these variables. Results are compared to predictive accuracy of BW and GA.

The model which analyzed GA and plasma protein simultaneously had an AUC of 0.873 (0.847–0.896) and the calibration at the HL goodness-of-fit test showed p = 0.39.

## Discussion

Our study has shown that low plasma protein values in the first day of life have a strong predictive ability for severe outcome in VLBWI. This result is consistent with our previous finding [9], which could be confirmed in another population, differing from the former for perinatal characteristics, rates of impaired outcome, and care practice. Moreover, the result was stable over time in this study cohort.

In the present study, the performance of protein levels was comparable to that of the CRIB and of the SNAPPE-II and greater than that of other validated severity scores, indicating that additional real time indices of neonatal illness, based on pathophysiological concepts, must be taken into account when considering factors influencing mortality and morbidity in vulnerable newborns.

The interest of this approach in predicting outcome is not new and it has been reported elsewhere: recently, two papers [17], [18] have highlighted the prognostic value of high early lactate levels, which in one case showed a mortality predictive ability similar to that of CRIB and CRIB-II in VLBWI [18]. One study from De Felice et al. [19], which investigated the predictive accuracy of CRIB and CRIB-II in VLBWI has underlined the need to seek new clinical risk-adjustment markers in high-risk newborns; these authors had previously demonstrated the relationship between physiological markers of early peripheral microcirculatory changes and neonatal illness severity scores in sick babies after birth [20], [21].

What our study adds, in relation to previous investigations in this domain, is the reproducible and remarkable prognostic value for the outcome, in very preterm infants, of plasma protein levels measured early in postnatal life. This biological variable has never been considered in establishing risk scores for neonatal disease severity [22], even when these scores were designed for measuring morbidity and mortality risk by mainly taking into account individual and physiological characteristics of the infants, as in the SNAP [23], in the SNAP-II or in the Neonatal Therapeutic Intervention Scoring System [24].

This assertion, far from representing a criticism of the above cited, well validated scores, simply aims to draw attention to a simple and easily available marker of physiological impairment, possibly influencing the in-hospital outcome of the vulnerable preterm baby.

The present study was not designed to investigate the pathophysiological basis of the association ‘plasma protein and in-hospital outcome’ of VLBWI, but we have previously described a significant positive correlation between low colloid oncotic pressure, low total protein levels and hypotension on day 1 of life in newborns with respiratory distress [25], and we hypothesised in one recent paper that low early protein levels may impair maintenance of intravascular volume and adequate blow flow to vital organs in critically ill premature babies [9].

Obviously, the hypothesis that low plasma protein levels may influence the cardiovascular adaptation and blood flow perfusion to organs in the immediate postnatal period must be rigorously established in future, prospective investigations.

It is worth noting here, that one of the factors associated with impaired outcome in VLBWI is the failure in cardiovascular adaptation after birth [26]–[28]. As we know, this variable is not among the items integrated in risk scores based on fixed covariates, such as the CRIB-II, and it is measured by parameters such as hypotension and urine output in scores conceived to capture the mortality risk also linked to physiological variables (SNAPP and SNAPP-II). Now, hypotension is a rough and delayed sign of impaired cardiovascular adaptation and in addition, more subtle disturbances in cardiovascular function display significant relationships with clinical illness severity [29], [30].

In our view, the search for new risk-indicators in sick preterm infants should take into account markers which could influence transitional circulation.

The limitations of our analysis include that, due to the study design, the collection window for protein value was variable around the first 12 hours after birth and extending up to 20 hours of life, so that both obstetrical and neonatal care measures during the early postnatal period may have influenced this parameter. Similarly, some data were recorded afterwards and this may have reduced the reliability and accuracy of the collected information. Finally, this report does not allow the conclusion that modifying plasma protein levels is necessary to improve outcome in VLBWI.

Nonetheless, our result supports the new finding that protein values represent a marker of abnormal physiological state which is strongly predictive of impaired outcome in preterm babies.

## Conclusion

The emphasis of this paper was to encourage neonatologists to pay close attention to plasma protein levels during postnatal transition in preterm babies, as these have a prognostic value for adverse in-hospital outcome of very premature, sick infants. Further investigations are needed to determine whether any benefit can be obtained by adding protein measurements to other validated illness scores for newborns. Moreover, our finding raises the interest in addressing the above issues regarding new markers of physiological derangement in early postnatal life, in future, prospective studies on VLBWI, thus providing additional insight into factors influencing mortality and morbidity for this vulnerable population.
